# Assessment of referrals to a multidisciplinary outpatient clinic for patients with back pain

**DOI:** 10.1179/2042618611Y.0000000021

**Published:** 2012-02

**Authors:** Sasha Gulati, Asgeir S Jakola, Ole Solheim, Sindre Gabrielsen, Pål A Godø, Bjørn Skogstad, Øystein P Nygaard

**Affiliations:** Department of Neurosurgery, St. Olavs Hospital, Trondheim, Norway; Department of Neuroscience, Norwegian University of Science and Technology, Trondheim, Norway; Department of Physical Medicine and Rehabilitation, St. Olavs Hospital, Trondheim, Norway; National Center for Spinal Disorders, Faculty of Medicine, Norwegian University of Science and Technology, Trondheim, Norway

**Keywords:** Manual therapy, Primary care, Spinal, Specialist care, Referrals

## Abstract

**Objective:**

Each year our multidisciplinary outpatient clinic for patients with back pain receives a large number of referrals from primary care physicians, manual physiotherapists, and chiropractors. We wanted to assess the quality of the referrals regarding the information provided about case history, clinical findings, and results from additional investigations.

**Material and methods:**

Two hundred and eighty six consecutive referrals received in the time period from 1 October 2008 to 1 March 2009 were reviewed. We investigated if the referrals contained 12 given items. The items were defined by consensus of the broad range of specialists working at the multidisciplinary outpatient clinic. All registered items were regarded as useful when assigning patients with a priority and appropriate caregiver at the outpatient clinic. The 12 items that our group felt were reflective of good referrals were information about occupational status, duration of symptoms, pain distribution, sensory symptoms, use of analgesics, alleviating and/or aggravating factors, systems enquiry (i.e. urination, bowel movements, and sleep), provided treatment, deep tendon reflexes, motor function, sensory examination, and radiculopathy tests (i.e. straight leg raise and/or foraminal compression test).

**Results:**

Two hundred and fifty six (89·5%) referrals were from primary care physicians, and the remaining came from physicians in internships, manual physiotherapists, and chiropractors. Six (2·1%) referrals contained all 12 items. On average each referral contained 5·95 items (95% CI: 5·66–6·25). Information about analgesics, sensory symptoms, systems enquiry, and alleviating and aggravating factors was most frequently missing. Information about provided treatment, motor function, deep tendon reflexes, clinical tests, and occupational status was included in about half of the referrals. In 27·3% of the referrals from primary care physicians information about clinical findings was missing. Referrals from manual physiotherapists contained statistically significant more information (9·67 items, 95% CI: 7·63–11·70) than from the other groups (*P*<0·001). The number of patients registered with each primary care physician did not affect the number of items in the referrals.

**Conclusion:**

Many of the referrals were inadequate. Inadequate referrals can lead to prolonged waiting time for examination and treatment. Referrals with relevant information about patient history and clinical findings are essential in order to assign patients with an appropriate caregiver at the outpatient clinic and to determine if and which diagnostic imaging findings are of clinical relevance.

## Background

Spinal disorders are prevalent in the population and are a significant cause of sick leave in Norway.^1^ Patients with spine symptoms have frequent contact with primary care providers and specialist health services. Patients with longstanding symptoms may present complex problems which are best addressed by a multidisciplinary team.^2^

Standardized referrals to somatic departments and medical specialists have been tested.^3^ Difficulties in reaching consensus regarding the common content of referrals were experienced as each medical field often requires specific information and additional investigations.^3^ As a result, standardized referrals have not been implemented in clinical practice. Previous studies have primarily focused on the frequency rate and necessity of referrals to specialist health care, and there are few studies assessing the quality of referrals.^4^^–^6 Good referrals probably improve patient care and make it easier to give patients correct priority.^3^,^7^ Thus, it is important that referrals contain relevant background information, information about the current problem, clinical findings, treatment provided, and what kind of additional investigations have been performed.

In general, all Norwegian residents are provided with a primary care physician. The number of patients registered with each primary care physician varies considerably and is publicly available. It has recently been debated if an upper limit of patients registered with each primary care physician should be introduced. Patients must be referred by a primary care physician in order to receive elective specialist health care services. Since 2006 manual physiotherapists and chiropractors are also allowed to refer patients to specialist health care services.

The primary aim of this study was to assess the quality of referrals from primary care givers (e.g. primary care physicians, physicians in primary care internships, manual physiotherapists, and chiropractors) received at the multidisciplinary outpatient clinic for patients with back pain at our hospital. We also wanted to investigate if the number of patients registered with primary physicians affected the quality of referrals. Further, we wanted to explore if there was any difference in the quality of referrals from different primary care givers.

## Material and Methods

In this prospective observational trial, we investigated 286 consecutive referrals for patients with spinal disorders between 1 October 2008 and 1 March 2009 received at the multidisciplinary outpatient clinic for patients with back pain, St Olavs University Hospital in Trondheim, Norway. The multidisciplinary outpatient clinic for patients with back pain is situated in a tertiary referral hospital serving a population of 630 000. Patients are only accepted after referral from primary care providers (e.g. primary care physicians, physicians in primary care internships, manual physiotherapists, and chiropractors). Based on the information provided in the referrals, patients were assigned with a treatment priority and to an assessment by one or more of the treatment providers working at the outpatient clinic. Treatment providers at the outpatient clinic include neurologists, neurosurgeons, specialists in physical medicine and rehabilitation, orthopedic surgeons, an anesthesiologist, a specialist in clinical pharmacology, a primary care physician, physiotherapists, and a manual therapist. The referrals were investigated with regards to 12 predetermined items defined by consensus of the specialists working at the multidisciplinary outpatient clinic. Everyone in the team was involved in daily treatment of patients with back pain. The 12 items that our group felt were reflective of good referrals were information about occupational status, duration of symptoms, pain distribution, sensory symptoms, use of analgesics, alleviating and/or aggravating factors, systems enquiry (i.e. urination, bowel movements, and sleep), provided treatment, deep tendon reflexes, motor function, sensory examination, and radiculopathy tests (i.e. straight leg raise and/or foraminal compression test). All 12 items were regarded as useful when assigning patients with a priority and appropriate caregiver at the outpatient clinic. In addition to these 12 items, we registered if any information about diagnostic imaging was provided. We registered sex and age for all primary care providers referring patients. For primary care physicians we registered the number of patients assigned to them, and if they were certified specialists. Only referrals from primary care providers were included, and referrals from other specialist health services were excluded. The Data Inspectorate in Norway approved registration and management of data. The study was approved by the Regional Committee for Medical Research Ethics for Health Region Mid-Norway. The need for informed consent was waived by the Norwegian Data Inspectorate and the Regional Committee for Medical Research Ethics for Health Region Mid-Norway. None of the referring clinicians knew about the study. Study protocols adhered to guidelines by the Helsinki Convention.

Statistical analyses were performed with SPSS version 16.0 (SPSS Inc., Chicago, IL, USA). Normal distribution was tested using *Q*–*Q* plots. For each referral we registered which of the 12 predetermined items were present (dichotomous variables: yes or no). The sum of items included in each referral was used in the Mann–Whitney *U* test to compare different treatment providers. Findings were considered statistically significant when *P* values were <0·05.

## Results

Among 286 referrals, 170 (59·4%) were from primary care physicians without specialist certification, 86 (30·1%) from primary care physicians with specialist certification, 10 (3·5%) from physicians in primary care internships, 11 (3·8%) from chiropractors and 9 (3·1%) from manual physiotherapists.

There were 152 (52·2%) female patients. The mean age was 50·2 and 45·1 years for patients and caregivers, respectively. There were 110 (37·2%) referrals from female primary caregivers.

For primary care physicians (excluding physicians in internships) the average number of patients registered with them was 1269 [95% confidence interval (CI): 1227–1310].

Six (2·1%) of the referrals contained all 12 items. The mean number of items in the referrals was 5·95 (95% CI: 5·66–6·25). Table 1 shows the proportion of referrals containing each of the recorded items from the patient history and clinical findings. In 272 (95·1%) referrals, pain distribution was provided. Information about the duration of symptoms and presence of sensory symptoms was present in 201 (70·3%) and 123 (43·0%) of the referrals, respectively. In referrals from primary care physicians (*n* = 256), 72·7% contained one or more items from the recorded clinical findings. Table 2 shows the proportion of referrals containing each of the recorded items from the patient history and clinical findings for each group of caregivers.

**Table 1:** Proportion of referrals containing each of the recorded items of clinical information

Clinical information	%
Pain distribution	95·1
Duration of symptoms	70·3
Treatment provided	57·3
Occupational status	53·8
Motor function	52·1
Radiculopathy tests	50·0
Sensory symptoms	43·0
Deep tendon reflexes	41·6
Alleviating/aggravating factors	39·9
Sensory examination	36·7
Use of analgesics	32·9
Systems enquiry	22·7

**Table 2:** Proportion (%) of referrals from each group of caregivers containing the recorded items from the patient history and clinical findings

Predefined parameters	PCP without specialist certification (*n* = 170)	PCP with specialist certification (*n* = 86)	Physicians in internship (*n* = 10)	Manual physiotherapists (*n* = 9)	Chiropractors (*n* = 11)
Duration of symptoms	68·8	69·8	90·0	100	54·6
Pain distribution	95·9	95·3	80·0	100	90·9
Sensory symptoms	38·2	47·7	30·0	77·8	63·6
Alleviating/aggravating factors	37·1	41·9	40·0	77·8	36·4
Treatment provided	54·1	61·6	50·0	100	45·4
Use of analgesics	31·2	38·4	40·0	33·3	9·1
Occupational status	54·7	55·8	30·0	77·8	27·3
Motor function	51·2	50·0	70·0	88·9	36·4
Systems enquiry	22·9	17·4	30·0	66·7	18·2
Sensory examination	37·6	29·1	60·0	88·9	18·2
Deep tendon reflexes	42·4	34·9	50·0	88·9	36·4
Radiculopathy tests	48·8	47·7	90·0	66·7	36·4

**Note:** PCP denotes primary care physicians.

Radiculopathy tests (i.e. straight leg raise and/or foraminal compression test) were described in 143 (50%) referrals. In 114 (39·9%) cases information about alleviating and/or aggravating factors was provided. There was information about treatment already provided in 163 (57·3%) referrals. Information concerning use of analgesics was described in 94 (32·9%) cases. Occupational status for patients between 16 and 67 years (*n* = 256) was provided in 126 (49·2%) referrals. Information about systems enquiry was given in 65 (22·7%) cases.

In 225 (78·7%) cases magnetic resonance imaging (MRI) was performed. Computed tomography (CT) and plain X-ray was performed in 48 (16·8%) and 67 (23·4%) cases, respectively. A combination of MRI and plain X-ray was performed in 46 (16·1%) cases. Both CT and MRI were performed in nine (3·1%) cases. MRI had been performed in 205 (77·1%) patients referred from primary care physicians. All patients referred from chiropractors and manual physiotherapists had undergone MRI investigations. In 15 (5·2%) cases no imaging studies had been conducted or ordered by the primary care giver.

Table 3 demonstrates the number of items included in the referrals from each group of primary care providers. Referrals from manual physiotherapists contained significantly more items compared to the others (*P*<0·001). There was no statistical significant difference between the other groups.

**Table 3:** Number of items included in the referrals from each group of primary care providers

Primary care provider	Number of predefined items (mean)	Standard deviation	95% confidence interval
Primary care physicians (certified specialists)	5·90	2·35	5·39–6·40
Primary care physicians (without specialist certification)	5·83	2·35	5·47–6·18
Physicians in internship	6·60	3·31	4·23–8·97
Chiropractors	4·73	2·90	2·78–6·68
Manual physiotherapists	9·67	2·65	7·63–11·70

The number of patients registered with primary care physicians (using the mean as a cut-off value) was not associated with differences in numbers of recorded items included in the referrals (*P* = 0·641).

## Discussion

This study shows that a large number of the referrals received were insufficient based on our a priori consensus of 12 important variables. Only six (2·1%) of the referrals contained all 12 registered items. Information about analgesics, sensory symptoms, systems enquiry, and alleviating and aggravating factors was most frequently missing. Information about provided treatment, motor function, deep tendon reflexes, clinical tests, and occupational status was included in about half of the referrals.

A recent study evaluated 198 referrals to a medical outpatient clinic.^8^ In this study, 63% of the referrals were found to be without shortcomings. Information concerning additional investigations and medications was most frequently absent. Another study from 2002 assessed the quality of referrals for patients older than 75 years admitted to an orthopedic and two medical wards.^9^ Information provided about medications was considered to be of low quality in 44% of the referrals. A British study from 1993 investigated the quality of general practitioner referrals to different outpatient departments.^10^ A total of 705 referrals, including 224 medical and 360 surgical, were analyzed. Errors or omissions concerning medications and past medical history were recorded in 26·2 and 28·2% of the referrals, respectively. Even though these studies are not directly comparable to ours, information concerning medications was absent in a higher proportion of referrals in our material. In our study, information concerning medications was omitted in 192 (67·1%) of the referrals. Information about the use of analgesics in particular is important when assessing the severity of a patient’s problems and might affect the priority the patient is given.

The patient history and physical examination are helpful when constructing a hypothesis, and are also important when deciding if image findings, clinical findings, and symptoms correlate. In addition, the physical examination is of great importance when establishing contact and trust between the patient and care provider. There is no doubt that information about clinical investigations is important for the management of patients. As this information was missing in 27·3% of referrals from the largest group, primary care physicians (*n* = 256), there seems to be great room for improvement. For the other groups it is difficult to draw any certain conclusions as their number of referrals is low in our study.

Information about radiculopathy tests was given in 50·0% of the referrals. Radiculopathy tests may help confirm a diagnosis and clarify the significance of image findings. The results of radiculopathy tests may assist in triage of patients and assigning the appropriate caregiver. However, it is difficult to determine the accuracy of radiculopathy tests and the value of these tests should be interpreted with caution.^11^,^12^

The majority of patients had undergone MRI investigations. MRI provides more information about soft tissue changes and changes in neural structures than CT.^13^ CT is well suited for assessing skeletal changes, but also represents considerable radiation risk.^14^ A combination of MRI and plain X-ray images was performed in 16·1% of patients. However, X-ray images provide little additional information and are superfluous in this context.^13^,^15^

The relevance of recording if information about systems enquiry was present is questionable. Information about decreased sleep or difficulties when going to the toilet might paint a clearer picture of the problems experienced. However, if there is suspicion of cauda equina syndrome, the patient should be admitted to hospital immediately.^15^

Referrals from manual physiotherapists contained statistically significant more information than from the other groups. This finding should be interpreted with caution as the number of referrals from manual physiotherapists and chiropractors in our study is relatively low. Due to the imbalance in the number of referrals received from each group, it is difficult to draw any certain conclusions regarding differences in the quality of referrals from different primary care givers. We did not take into account that a few of the primary care providers may have supplied us with more than one referral (i.e. if one primary care physician referred two different patients), and this further complicates comparison of the occupational groups. The number of patients registered with each primary care physician did not affect the number of items in the referrals. However, the variation in number of patients registered with each primary care physician was low in our study.

A limitation of this study was the considerable variation in the number of referrals from each occupational group, and this complicates the interpretation of the statistical analysis. The parameters registered are not validated in any way, and they are therefore presented primarily in a descriptive manner. The selection of items used in this study is debatable. The list of registered items is not exclusive, and we acknowledge that the different primary care providers may use different clinical tests in their daily practice. The 12 items were chosen on what we consider should be a common platform for all the primary care providers. Moreover, the selected items also help in determining how the patient history and clinical information correlate with image findings. This is especially important when deciding if a patient should receive a surgical assessment or not at the outpatient clinic. All registered items provide relevant background information for the specialists evaluating the patients at the outpatient clinic, and it is also reassuring to see if clinical findings concur and are reproducible with those described by the referrer as many patients have complex spinal disorders.

The threshold for what qualifies as a good referral is also debatable. Compared to previous studies, the referrals in our study seem to be more insufficient. It is difficult to determine if this is due to strict assessment criteria or actually poorer referrals. It seems that the specialized health services have a pronounced desire for details concerning the specific conditions under their care. The amount of information in referrals requested by specialist health services, represent a challenge for primary care providers. It has been suggested that electronic health journals could be designed to make reminders for making better and more disease specific referrals (i.e. tools to aid in decision making, assuring updated medication lists appear automatically in referrals, suggesting relevant clinical assessments).^16^ Such systems might allow a better flow of information between providers of care. The documented of lack of information in the referral letters in the present study may be the first step in improving the quality of the referrals, and may be used in a dialogue between multidisciplinary outpatient clinics for back pain and primary care givers.

## Conclusion

This study shows that many of the referrals were inadequate in the sense that basic information was missing. Good referrals are essential to give the patients a correct priority and assign them to the correct caregiver. The number of patients registered with primary care physicians did not influence the quality of referrals. Referrals from manual physiotherapists contained statistically significant more information than from the other groups. The amount of relevant information in primary care providers’ referral letters needs improvement. For primary care providers this represents yet another challenge.
